# Callus massage after distraction osteogenesis using the concept of lengthening then dynamic plating

**DOI:** 10.1007/s11751-015-0233-3

**Published:** 2015-09-04

**Authors:** Leonard Grünwald, Stephan Döbele, Dankward Höntzsch, Theddy Slongo, Ulrich Stöckle, Thomas Freude, Steffen Schröter

**Affiliations:** Department of Traumatology and Reconstructive Surgery, BG Trauma Center Tübingen, Eberhard Karls University Tübingen, Schnarrrenbergstr. 95, 72076 Tübingen, Germany; Inselspital, Bern University Hospital, Bern, Switzerland

**Keywords:** Dynamic locking screw, Distraction osteogenesis, Multiplanar deformity, Taylor spatial frame, Lengthening then plating

## Abstract

Correction of complex deformities is a challenging procedure. Long-term wearing of a fixator after correction and lengthening are inconvenient and has a high rate of complication. The goals of the surgical treatment in the presented case were: (1) correction of the deformity and lengthening of the left leg by the Taylor spatial frame (TSF, Smith and Nephew, Marl, Germany); (2) reduction in the time the patient wears the TSF by changing the fixation system to a plate (lengthening then plating—LTP) and using a locking compression plate in conjunction with the 5.0 dynamic locking screws in order to accelerate bone healing.

## Introduction

Correction of complex deformities using a ring fixator is a common technique, but treatment by distraction osteogenesis for deformity correction is challenging. A long duration with a ring fixator can cause patient dissatisfaction [1]. Complications including pin-track infection are frequent [2, 3]. Hexapod ring fixators are rigid and allow a minimum of interfragmentary movement only [4]. Claes et al. [5] and Goodship et al. [6] have described the influence of interfragmentary movement on healing in a bone defect model in sheep. Increased healing with movement between 0.2 and 1.0 mm was identified [7]. However, Lujan et al. [8] described more bone formation at the medial side in distal femur fractures after fixation with a LCP plate. The dynamic locking screw (DLS, DePuySynthes, Switzerland) concept offers 0.35-mm relative motion between the screw head and thread due to its pin–sleeve design. The use of DLS reduces the stiffness of the plate–screw interface and thus increases the interfragmentary motion at the near cortical side without altering the advantages of angular stability and the strength [9].

## Case report

**Fig. 1:**
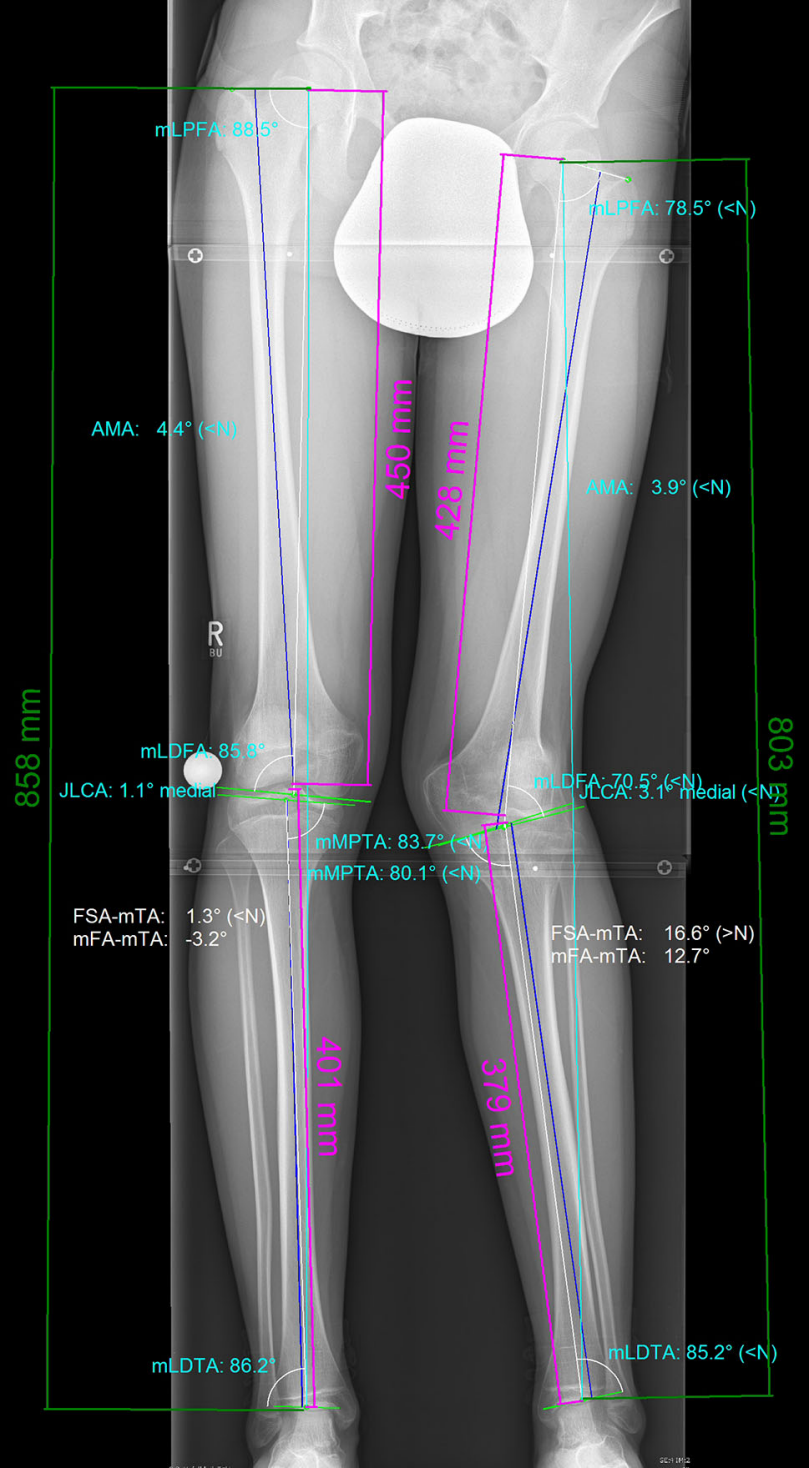
Preoperative full-weight-bearing long-leg radiograph. Analysis of the deformity with the planning software mediCAD (Hectec, Germany), angles according to Dror Paley. Measurements are in Table 1

**Fig. 2:**
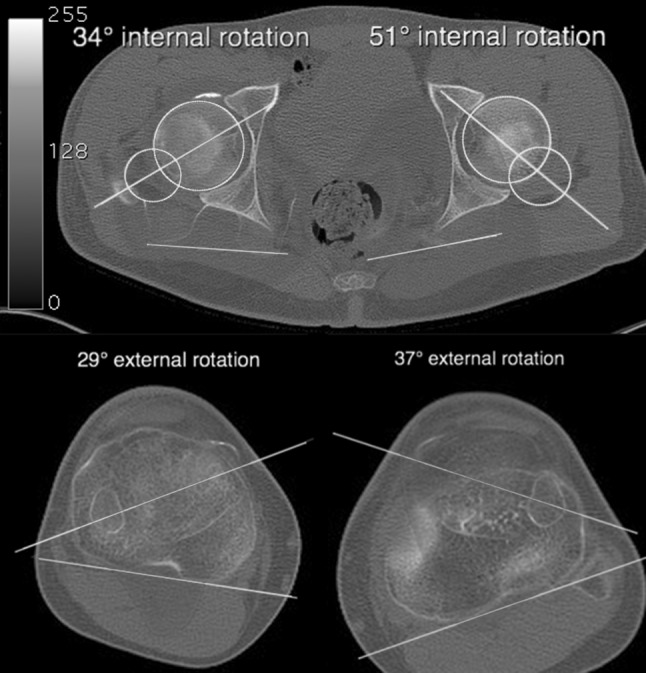
Preoperative CT scan. Analyses of the rotational deformity of the femur and the tibia using Osirix MD (Switzerland). Method of measurement according to Waidelich [10]

A 10-year-old patient sustained an anterior cruciate ligament (ACL) tear during school sport. A transepiphyseal ACL reconstruction using hamstring tendon was performed in July 2007. In 2009, it was recognised that an increasing deformity of the injured limb produced symptoms particularly pain during sports. Physical examination showed a valgus deformity with a leg length discrepancy of 5.5 cm. The range of motion (ROM) of the left knee was extension/flexion of 15°-0°-150°. The patella was located on the lateral femoral condyle due to internal rotation of the distal femur. When the knee was flexed to 60°, the patella slid back into the trochlea groove. There was no pain in the lateral or medial compartment of the knee. The MRI showed a bony bridge in the growth plate with an intact ACL and posterior cruciate ligament (PCL). Full-weight-bearing long-leg radiographs and anteroposterior and lateral knee views were performed. The valgus deformity was confirmed (Fig. 1). All joint angles were measured (Table 1) using mediCAD (Hectec, Germany). Additionally, a CT scan was performed, and rotational deformity measured was using Osirix MD (Pixmeo SARL, Switzerland) (Fig. 2).

**Table 1:** *Preoperative* analyses of the deformity

	Right leg	Left leg
mTFA	−3.2°	12.7°
MPTA	83.7°	80.1°
mLDFA	85.8°	70.5°
Femoral rotation (CT)	−34°	−51°
Tibial rotation (CT)	+29°	+37°
Tibial slope	8.5°	−16.4°
TT–TG distance (CT, cm)	1.16	2.15
Length femur (mm)	450	428
Length tibia (mm)	401	379
Leg length (mm)	858	803

*mTFA* mechanical tibiofemoral angle, *MPTA* medial proximal tibial angle, *mLDFA* mechanical lateral distal femur angle, femoral torsion was measured according to Waidelich [10]. Tibial slope according to Amendola [11]. *TT*–*TG* Tuberositas Tibiae–Trochlea groove distance

**Fig. 3:**
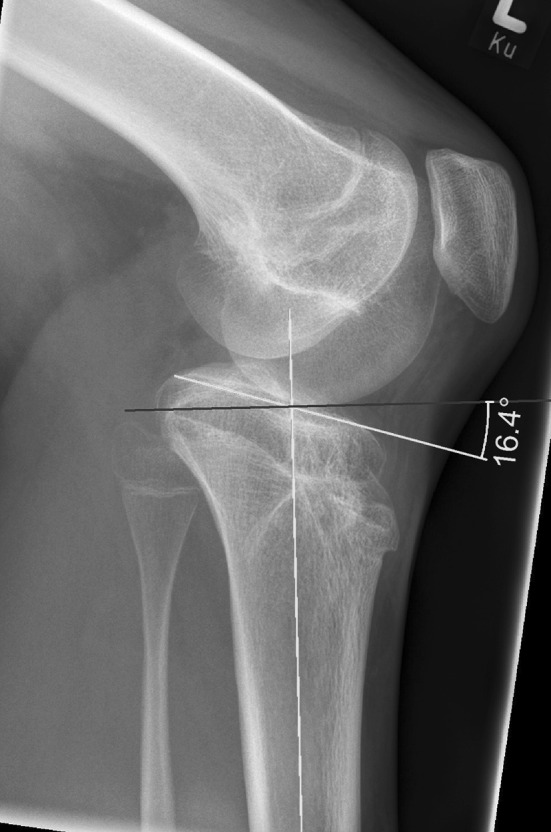
Preoperative knee lateral view. Tibial slope according to Amendola [11]

The deformity of the left leg was measured to consist of a femoral rotation discrepancy of 17° compared to the right femur, a valgus deformity with a mechanical tibiofemoral angle (mTFA) of 12.7°, a negative tibial slope of −16.4° (Fig. 3), and a leg length discrepancy of 5.5 cm, with the left leg shorter than the right.

The goals of the surgical treatment were: (1) correction of the deformity and lengthening of the left leg by a hexapod ring fixator (TSF, Smith and Nephew, Marl, Germany); (2) reducing the time in external fixation by changing the fixation system to a plate (lengthening then plating—LTP) [12]; and (3) using a locking compression plate in conjunction with the 5.0 dynamic locking screws (DLS) [13] in order to accelerate bone healing.

Two hexapod fixators were used, one for the femur and one the tibia. Both constructs were constructed in advance to reduce surgical procedure time and the frame diameters checked to avoid impingement of soft tissue. The proximal ring of the femoral TSF was fixed with a wire in the anterior third of the frame allowing for sufficient clearance of soft tissue in the posterior part of the thigh. An additional wire was passed at an angle of 45° to the first one followed by two Schanz screws to achieve more stability in the construct. The mounting parameters were measured from the proximal frame. The distal ring of the femoral fixator was fixed at an angle approximate to the measured deformity and was, as such, parallel to the aLDFA. The struts were then inserted and the length measurements of the struts noted. Two of the struts were removed temporarily to allow for the osteotomy of the distal femur performed using a Gigli saw.

**Fig. 4:**
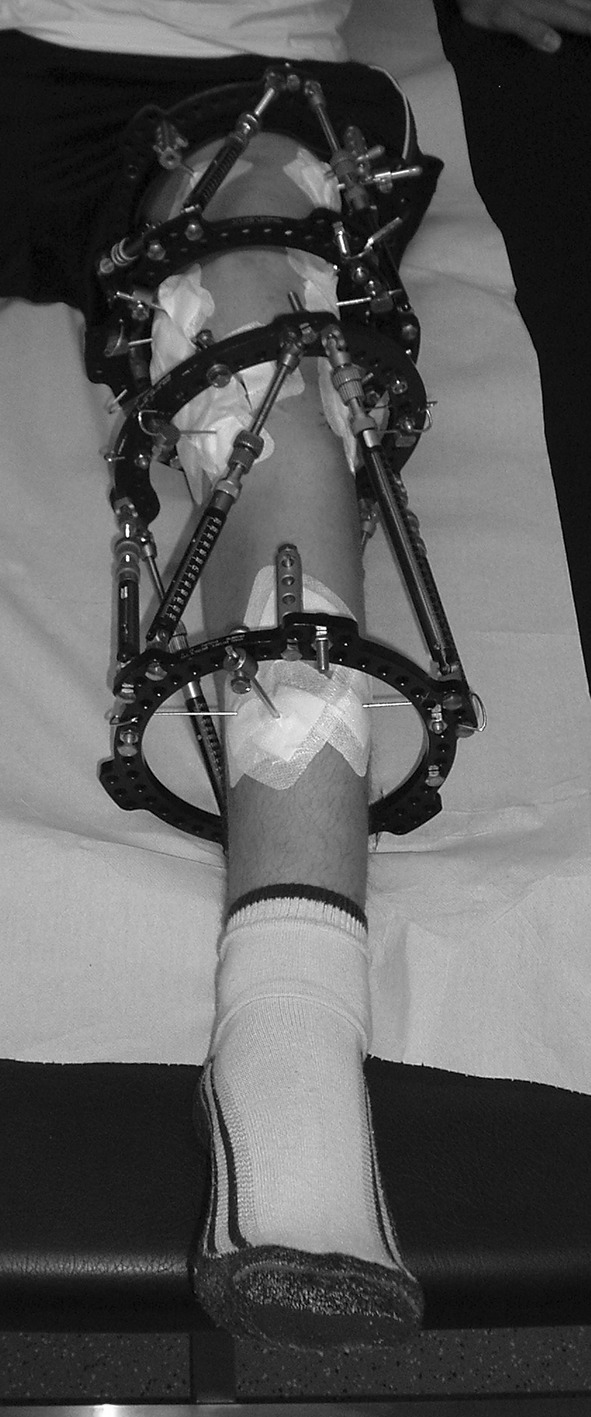
Clinical picture of the mounted double TSF, 4 weeks postoperatively

The tibial TSF was mounted in the same manner. The proximal frame was fixed parallel to the MPTA and to the tibial slope and this position used for the mounting parameters. Additionally, the fibula was fixed to the tibia with a K-wire distally. After connecting the rings with struts to reflect the measured deformity, an osteotomy distal to the tibial tuberosity and a distal fibula osteotomy were performed (Fig. 4).

A latency period of 7 days was applied and correction started according to the protocol derived from the Web-based software for the TSF. Continuous correction by distraction osteogenesis can lead to a decreasing ROM despite physiotherapy. At the point lengthening was stopped, physical examination documented a maximum ROM at the knee of 40° flexion. To reduce the duration of the fixator and its interference with progress with physiotherapy, a second surgery was performed 3 months after the first and in accordance with the strategy of lengthening then plating. The second surgery was started with removal of the fixator and the stability at the osteotomy tested intraoperatively. Under fluoroscopy control, the osteotomy of the femur was deemed stable. In contrast, the distraction callus at the tibia was flexible, and the final correction of the MPTA and the tibial slope was possible under anaesthesia. A 4-cm incision was performed at the proximal tibial head medial, and a nine-hole 5.0 LCP T-plate was subcutaneously inserted using the minimal invasive osteosynthesis (MIO) technique. Distally a further incision of 6 cm was performed. For proximal fixation, three 5.0 DLS were used and four 5.0 LS were inserted distally. Aftercare with physiotherapy and 20-kg partial weight bearing was performed combined with continuous passive motion machine (CPM). After 2 weeks, partial weight bearing was changed to full weight bearing.

**Fig. 5:**
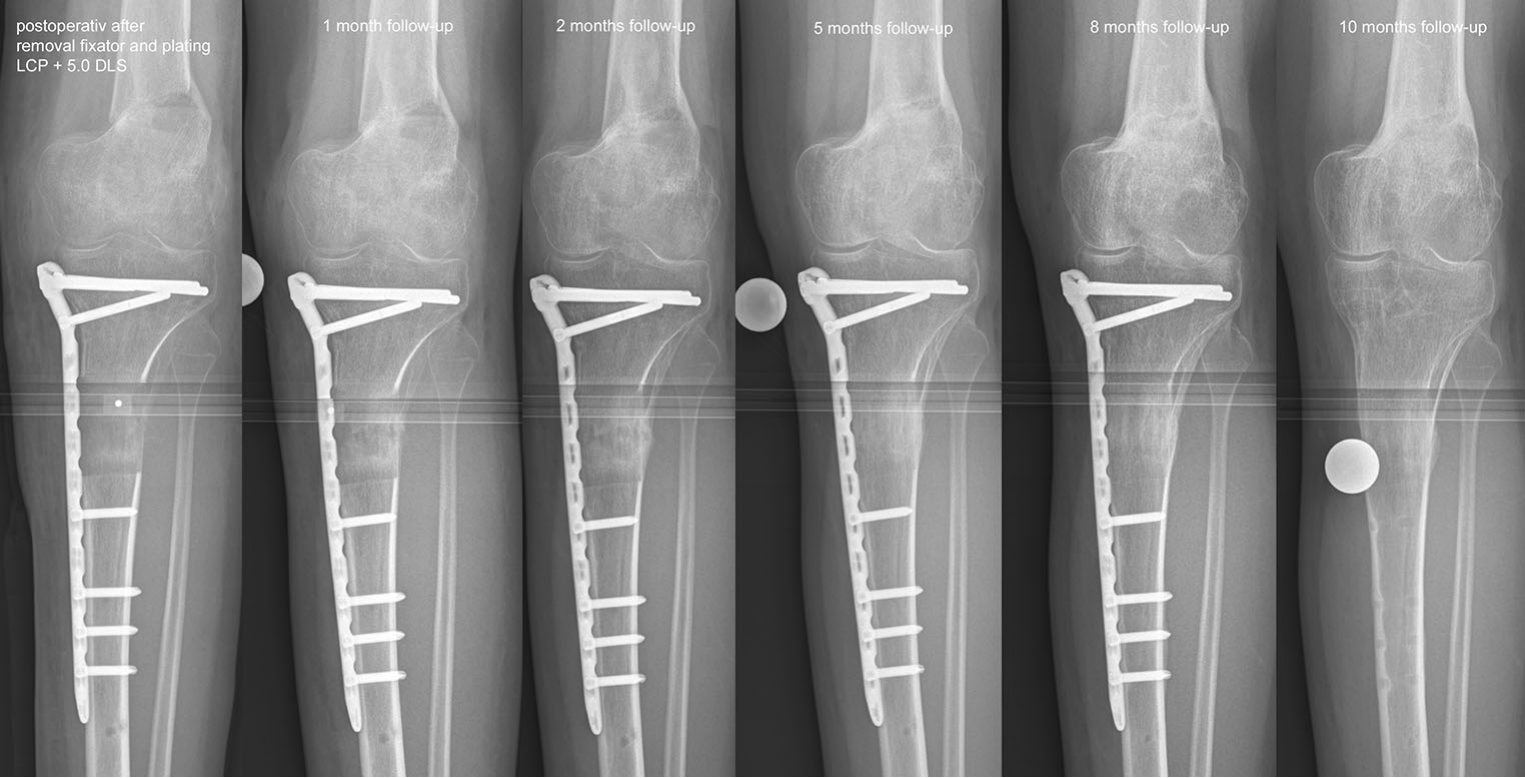
Follow-up radiographs. Osteosynthesis with a nine-hole LCP in conjunction with three DLS 5.0 proximal and four LS 5.0 distal. Continuous bone healing. After 2 months, criteria for a biomechanically stable situation are visible. After 5 months, complete homogenous bony structures are visible

**Fig. 6:**
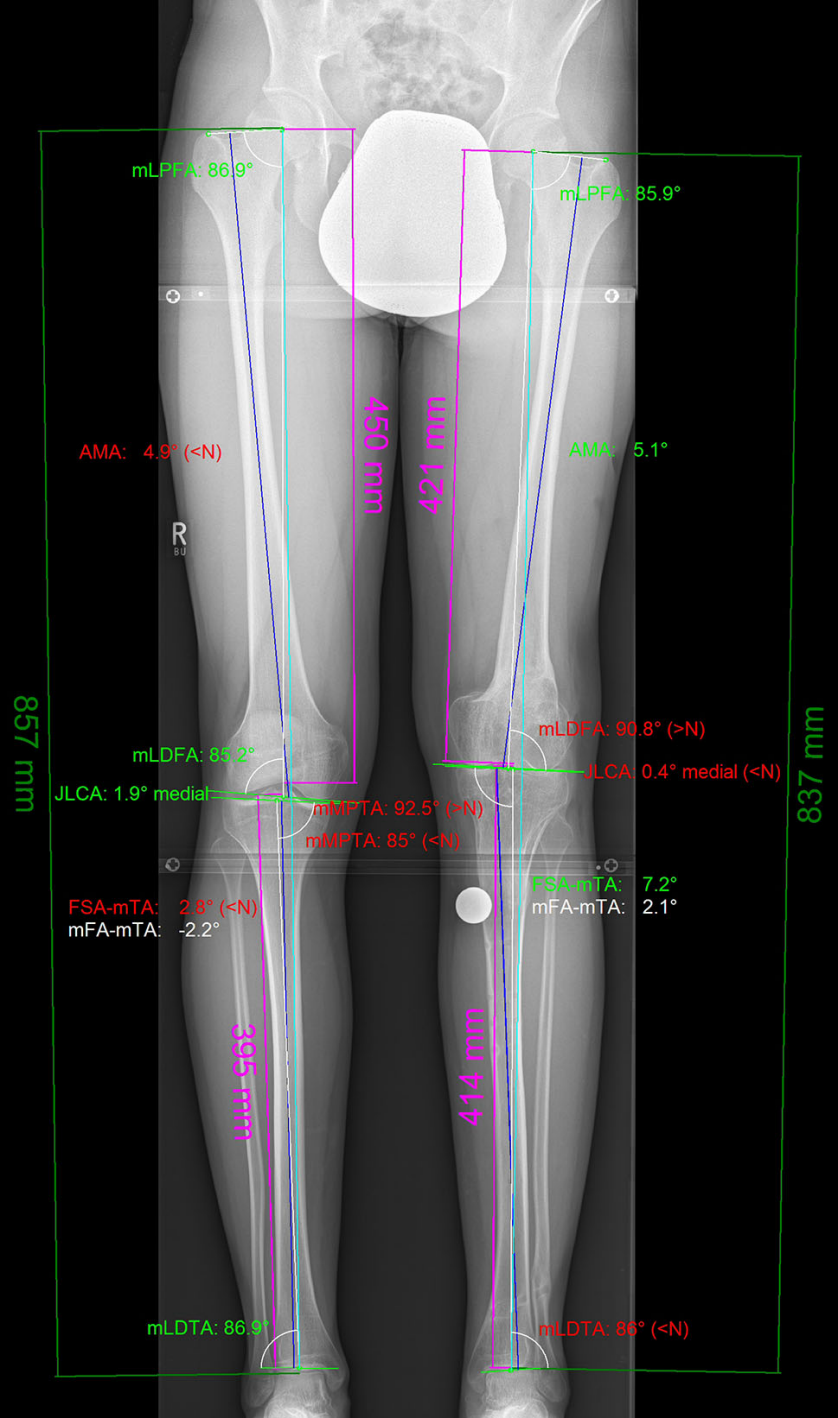
Postoperative full-weight-bearing long-leg radiograph. Analysis of the deformity with the planning software mediCAD (Hectec, Germany), angles according to Dror Paley. Measurements are in Table 2

**Fig. 7:**
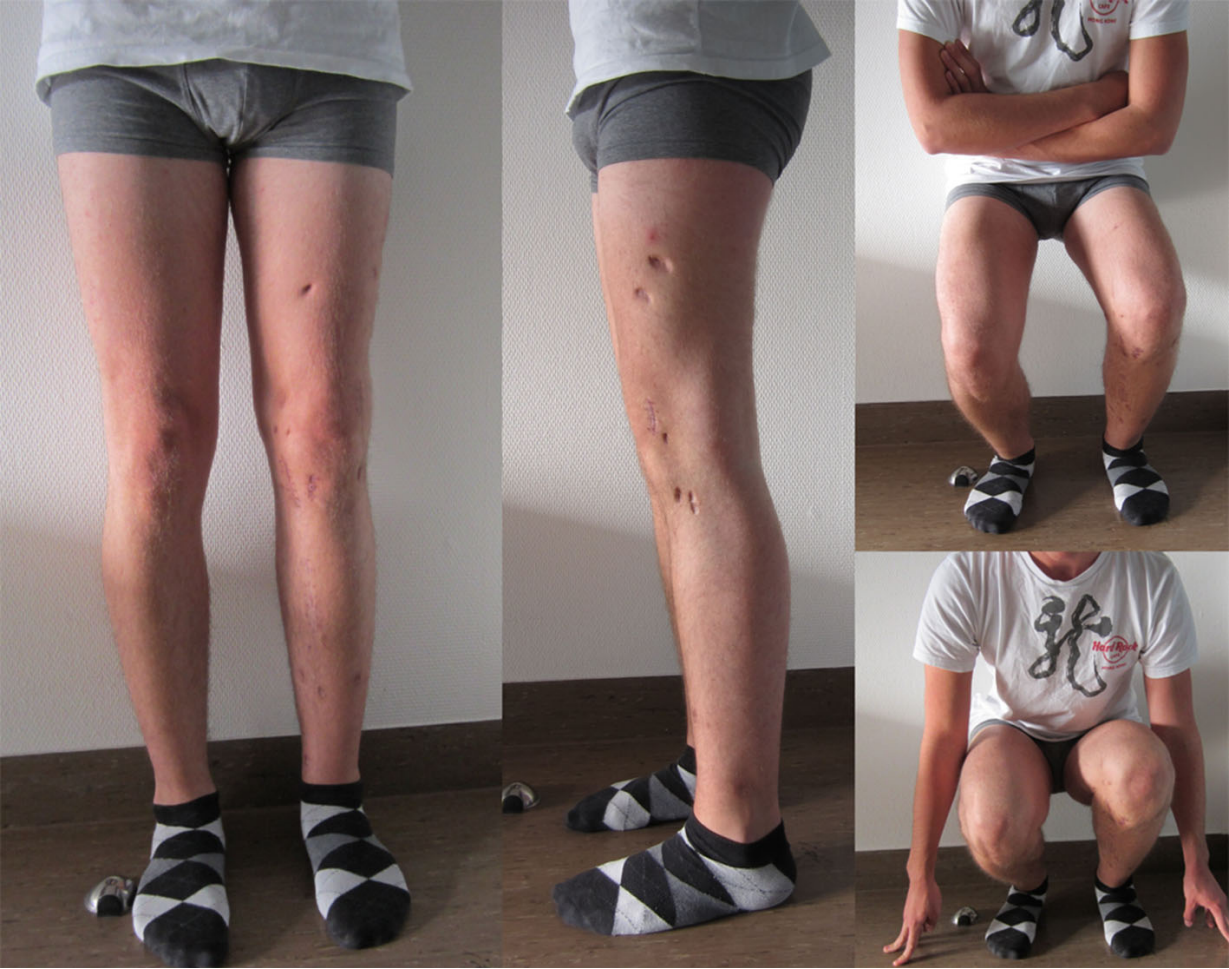
Clinical pictures at the final follow-up after 10 months

At 2 months postoperatively, the radiographs showed a complete maturity of the distraction callus and, at after 5 months, a homogenous structure of the bone (Fig. 5). The bone healing index (BHI) was 1.1 months/cm. After 8 months, the plate was removed as well as the K-wire from the distal tibiofibular joint. Removal of the DLS 5.0 was without complications. A final physical examination in September 2012 showed good alignment of the left leg (Fig. 6; Table 2), and the ROM at the right knee joint extension/flexion was 0°/0°/130° (Fig. 7). The patient had started moderate physical activity, such as cycling, and reported no pain. Low instability of his ACL was noted as the only pathological finding.

**Table 2:** Postoperative analyses of the deformity

	Right leg	Left leg
mTFA	−2.2°	2.1°
MPTA	85°	92.5°
mLDFA	85.2°	90.8°
Tibial slope	8.5°	2.5°
Length femur (mm)	450	421
Length tibia (mm)	395	414
Leg length (mm)	857	837

*mTFA* mechanical tibiofemoral angle, *MPTA* medial proximal tibial angle, *mLDFA* mechanical tibiofemoral angle. Tibial slope according to Amendola [11]

## Discussion

The presented technique using the strategy of lengthening followed by dynamic plating with the new dynamic locking screw (DLS) 5.0 which allows for symmetrical motion and callus modulation. The callus ‘massage’ is thought to produce early maturity of the distraction callus. Using this strategy of lengthening then dynamic plating with the MIO technique, most of the aspects of bone healing are respected.

Anterior cruciate ligament tears in immature patients are a controversial issue. The results of conservative treatment can be followed by continuing instability, problematic meniscal tears, osteochondral fractures, and potential subsequent osteoarthritis. Reports of ACL reconstruction using anatomical placement with transepiphysial tunnels and anchorage far away from the growth plate resulted in a good clinical outcome [14] but when used in the skeletally immature may produce growth arrest or growth disturbance [15, 16]. This can lead to a leg length discrepancy with angular deformity. This case report describes a solution for the development of deformity and leg length discrepancy after such a growth arrest. Different treatment strategies are available: Instead of a ring fixator, a double-level osteotomy at the distal femur and proximal tibia with bone allograft is a possible solution, but this will not address the leg length discrepancy. Gradual correction with a ring fixator is a well-established treatment for this problem. Whilst hexapod ring fixators offer correction in all six degrees of freedom [17], deformity analysis should precede surgery, and the actual treatment remains challenging. Web-based software programmes that are supplied with hexapod ring fixators calculate from the mounting parameters entered by the orthopaedic surgeon; these need to be accurate. Complication like stiffness of the knee is frequent during lengthening of the leg [18, 19]. Pin infections have been described by many authors, and the fixator is inconvenient for the patient [12]. Different solutions have been suggested for these complications. Physiotherapy with the fixator can be difficult; bending the knee produces stress across the tensioned wires, especially if the wires or Schanz screws are located close to a tendon or transfix muscle or the iliotibial tract. This problem is related to the position of the Schanz screws and wires as they pass obliquely through the quadriceps in a direction from medial and lateral. Additionally, the wire on the distal femoral ring passes through the capsule of the knee joint but was required to maintain sufficient stability. The aim of this treatment strategy was early removal of the fixator to facilitate progress with physiotherapy. Nailing after lengthening [20] and lengthening then plating [12] have been described to enable this objective. Concerns over a higher rate of deep infection have not been fully realised [12, 20]. In the present case, nailing was deemed to be technically demanding in view of the mounted TSF because at the first surgery a change of the fixator to plating was not envisaged. Although higher complication rates of deep infection are not reported with this technique, we advocate using this strategy only if the patient has no serious infections related to the Schanz screws or wires. Lengthening then plating has complications such as breakage of the implant when there is delayed maturity of the distraction callus [12]. To reduce this risk the bone healing should be accelerated. With the new DLS concept, dynamization through the plate is possible. Symmetrical motion under the plate and at the far cortex [21] results in callus modulation. With a maximum motion of 0.35 mm in the DLS 5.0, the results and recommendations from sheep studies by Claes et al. [7] seemed to be realised. This principal appears a logical concept to accelerate bone healing. The observed BHI of our patient with 1.1 months/cm is lower than the BHI of 2.1 months/cm from LTP without the DLS [12]. Limitations in this evaluation and comparison with other studies lie in the definition of the BHI. Generally, three visible cortices in the regenerate column on two orthogonal plain radiographs are defined as the end point. However, agreement for a reliable method to measure the bone healing in distraction callus is not yet available.

## Summary

We present a case of correction of leg length discrepancy and deformity using the lengthening then plating strategy. Internal fixation was performed using dynamic locking screws in a plate which allowed for callus modulation and a low bone healing index of 1.1 months per centimetre. This appears to facilitate a rapid consolidation of the regenerate column and may assist in preventing failure of the plate before consolidation of the callus.

## References

[1] Fischgrund J, Paley D, Suter C (1994). Variables affecting time to bone healing during limb lengthening. Clin Orthop Relat Res.

[2] Al-Sayyad MJ (2011). Taylor spatial frame in the treatment of neglected fractures. J Child Orthop.

[3] Sun XT, Easwar TR, Manesh S, Ryu JH, Song SH, Kim SJ, Song HR (2011). Complications and outcome of tibial lengthening using the Ilizarov method with or without a supplementary intramedullary nail: a case-matched comparative study. J Bone Joint Surg Br.

[4] Khurana A, Byrne C, Evans S, Tanaka H, Haraharan K (2010). Comparison of transverse wires and half pins in Taylor spatial frame: a biomechanical study. J Orthop Surg Res.

[5] Claes LE, Wilke HJ, Augat P, Rubenacker S, Margevicius KJ (1995). Effect of dynamization on gap healing of diaphyseal fractures under external fixation. Clin Biomech (Bristol, Avon).

[6] Goodship AE, Kenwright J (1985). The influence of induced micromovement upon the healing of experimental tibial fractures. J Bone Joint Surg Br.

[7] Claes LE, Heigele CA, Neidlinger-Wilke C, Kaspar D, Seidl W, Margevicius KJ, Augat P (1998). Effects of mechanical factors on the fracture healing process. Clin Orthop Relat Res.

[8] Lujan TJ, Henderson CE, Madey SM, Fitzpatrick DC, Marsh JL, Bottlang M (2010). Locked plating of distal femur fractures leads to inconsistent and asymmetric callus formation. J Orthop Trauma.

[9] Dobele S, Horn C, Eichhorn S, Buchholtz A, Lenich A, Burgkart R, Nussler AK, Lucke M, Andermatt D, Koch R, Stockle U (2010). The dynamic locking screw (DLS) can increase interfragmentary motion on the near cortex of locked plating constructs by reducing the axial stiffness. Langenbecks Arch Surg.

[10] Waidelich HA, Strecker W, Schneider E (1992). Computed tomographic torsion-angle and length measurement of the lower extremity. The methods, normal values and radiation load. RoFo.

[11] Amendola A, Rorabeck CH, Bourne RB, Apyan PM (1989). Total knee arthroplasty following high tibial osteotomy for osteoarthritis. J Arthroplasty.

[12] Harbacheuski R, Fragomen AT, Rozbruch SR (2012). Does lengthening and then plating (LAP) shorten duration of external fixation?. Clin Orthop Relat Res.

[13] Freude T, Schroter S, Kraus TM, Hontzsch D, Stockle U, Dobele S (2013). Dynamic locking screw 5.0—first clinical experience. Z Orthop Unfall.

[14] Aichroth PM, Patel DV, Zorrilla P (2002). The natural history and treatment of rupture of the anterior cruciate ligament in children and adolescents. A prospective review. J Bone Joint Surg Br.

[15] Chotel F, Henry J, Seil R, Chouteau J, Moyen B, Berard J (2010). Growth disturbances without growth arrest after ACL reconstruction in children. Knee Surg Sports Traumatol Arthrosc.

[16] Kocher MS, Saxon HS, Hovis WD, Hawkins RJ (2002). Management and complications of anterior cruciate ligament injuries in skeletally immature patients: survey of the Herodicus Society and the ACL Study Group. J Pediatr Orthop.

[17] Seide K, Wolnack J, Weinrich N, Jurgens C (2002). Theory and software of the hexapod external fixator. Biomed Tech (Berl).

[18] Martin BD, Cherkashin AM, Tulchin K, Samchukov M, Birch JG (2013). Treatment of femoral lengthening-related knee stiffness with a novel quadricepsplasty. J Pediatr Orthop.

[19] Iacobellis C, Berizzi A, Aldegheri R (2010). Bone transport using the Ilizarov method: a review of complications in 100 consecutive cases. Strategies Trauma Limb Reconstr.

[20] Rozbruch SR, Kleinman D, Fragomen AT, Ilizarov S (2008). Limb lengthening and then insertion of an intramedullary nail: a case-matched comparison. Clin Orthop Relat Res.

[21] Dobele S, Horn C, Eichhorn S, Buchholtz A, Lenich A, Burgkart R, Nussler AK, Lucke M, Andermatt D, Koch R, Stockle U (2010). The dynamic locking screw (DLS) can increase interfragmentary motion on the near cortex of locked plating constructs by reducing the axial stiffness. Langenbecks Arch Surg.

